# Supplementary material for: Optimization of Acute Kidney Injury (AKI) Time Definitions Using the Electronic Health Record: A First Step in Automating In-Hospital AKI Detection
Source: J Clin Med. 2021 Jul 27;10(15):3304. doi: 10.3390/jcm10153304 (PMC8347988; doi:10.3390/jcm10153304)
Supplement: Supplementary file 1 [file jcm-10-03304-s001.zip › jcm-1285472-supplementary.pdf]

Supplemental Materials

Optimization of acute kidney injury (AKI) time definitions using the electronic health record: A first step in automating in-hospital AKI detection

Joshua T. Swan, PharmD, MPH (co-primary author); Linda W. Moore, PhD (co-primary author); Harlan G. Sparrow, MBA, MA; Adaani E. Frost, MD; A. Osama Gaber, MD; Wadi N Suki, MD

Corresponding author: Joshua T. Swan, PharmD, MPH; swan.joshua@gmail.com

Supplemental Material Table of Contents

Text S1. Applied examples for observation windows and precision levels

Figure S1. Schema for observation window expansions

Figure S2. Histogram of serum creatinine assessments by hour of day (n=673,011)

Figure S3. Days of hospital care before and after detection of AKI as the observation window increases from 24 to 172 hours (n=124,566 survivors only)

Figure S4. Days of hospital care before and after detection of AKI using only criterion 1a (absolute increase of 0.3 mg/dL) as the observation window increases from 24 to 172 hours (n=126,367)

Figure S5. Days of hospital care before and after detection of AKI using only criterion 1b (50% relative increase) as the observation window increases from 24 to 172 hours (n=126,367)

Figure S6. Adjusted odds ratio of in-hospital mortality by narrowest observation window using only criterion 1a (absolute increase of 0.3 mg/dL)

Figure S7. Adjusted odds ratio of in-hospital mortality by narrowest observation window using only criterion 1b (50% relative increase)

Table S1. Association between AKI and length of stay (n=126,367)

Table S2. Association between AKI and in-hospital mortality (n=126,367)

Text S1. Applied examples for observation windows and precision levels

The following examples demonstrate how precision levels were operationalized for a 1-day observation window. Assume that a patient had 4 consecutive SCr assessments during the hospital stay, which were drawn on Monday at 4:00 AM (#1), Monday at 5:00 PM (#2), Tuesday at 3:30 AM (#3), and Tuesday at 6:00 AM (#4). For the calendar day precision level, AKI could only be detected by evaluating 2 pairs of SCr assessments (#1 vs. #2 and #3 vs #4). For the 24-hour precision level, AKI could be detected using 4 pairs of SCr assessments (#1 vs #2, #1 vs #3, #2 vs #3, and #3 vs #4). For the 28-hour precision level, AKI could be detected by evaluating 6 pairs of SCr (#1 vs #2, #1 vs #3, #1 vs #4, #2 vs #3, #2 vs #4, and #3 vs #4). As apparent from the example above, precision levels impact the quantity of SCr comparisons that can be used to detect AKI.

Figure S1. Schema for observation window expansions

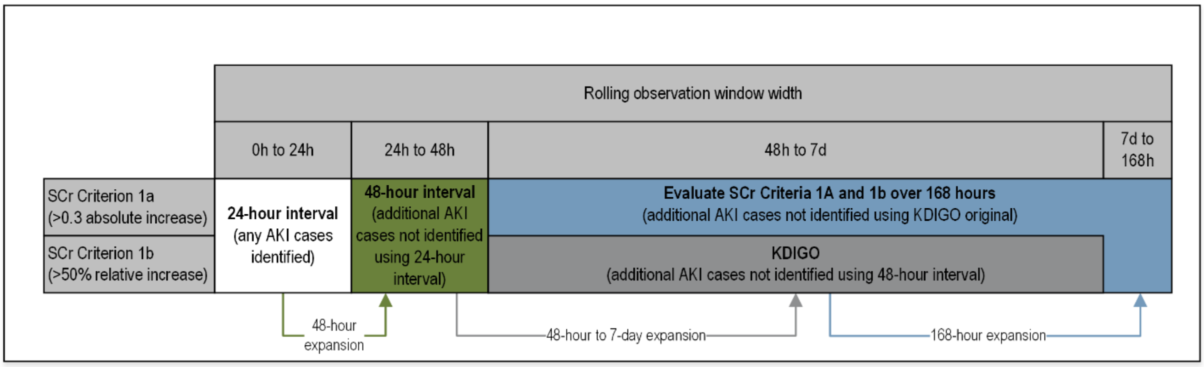

First, the analysis identified all cases of AKI that could be detected using a 24-hour observation window for SCr criteria A and B. Second, additional cases of AKI were detected by expanding observation windows for SCr criteria A and B from 24 to 48 hours (48-hour expansion). Third, additional cases of AKI were detected by expanding the observation window for SCr criterion B to 7 days (48-hour to 7-day expansion). Last, SCr criteria A was expanded from 48 hours to 168 hours and SCr criterion B was clarified from 7 days to 168 hours (168-hour expansion)

Figure S2. Histogram of serum creatinine assessments by hour of day (n=673,011)

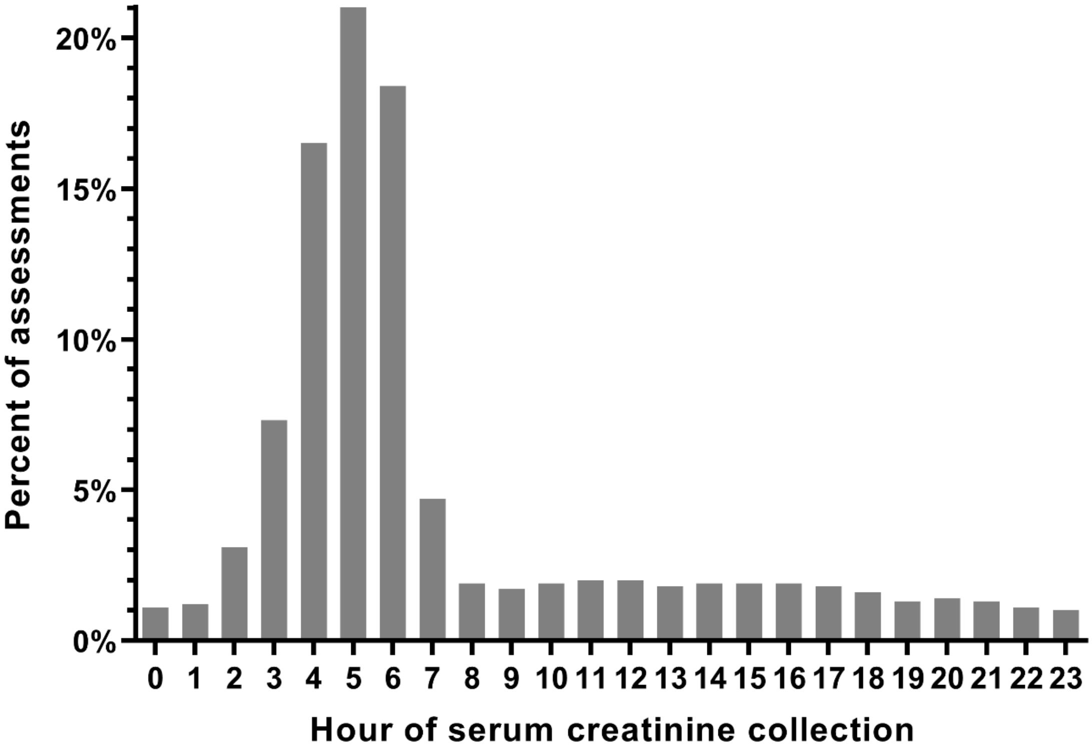

This histogram shows data for 673,011 serum creatinine (SCr) assessments collected from 2012 to 2014 for patients included in this study. The height of the bar shows the proportion of SCr assessments collected during each hour of the day, where "0" represents 12:00 AM to 12:59 AM. Sixty-eight percent of measurements were collected between the hours of 3:00 AM and 7:59 AM.

Figure S3. Days of hospital care before and after detection of AKI as the observation window increases from 24 to 172 hours (n=124,566 survivors only)

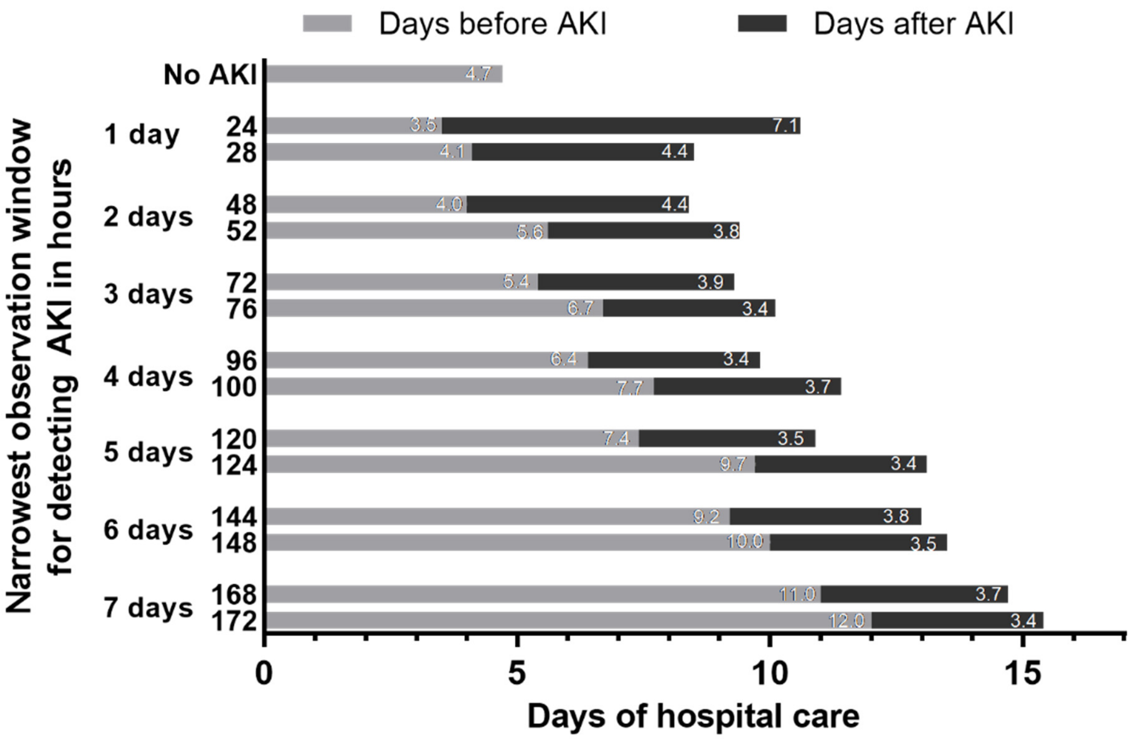

AKI acute kidney injury.

Among survivors, AKI prolonged the hospital stay 3-4 days after AKI detection, with 7 days for the 24-hour window.

Figure S4. Days of hospital care before and after detection of AKI using only criterion 1a (absolute increase of 0.3 mg/dL) as the observation window increases from 24 to 172 hours (n=126,367)

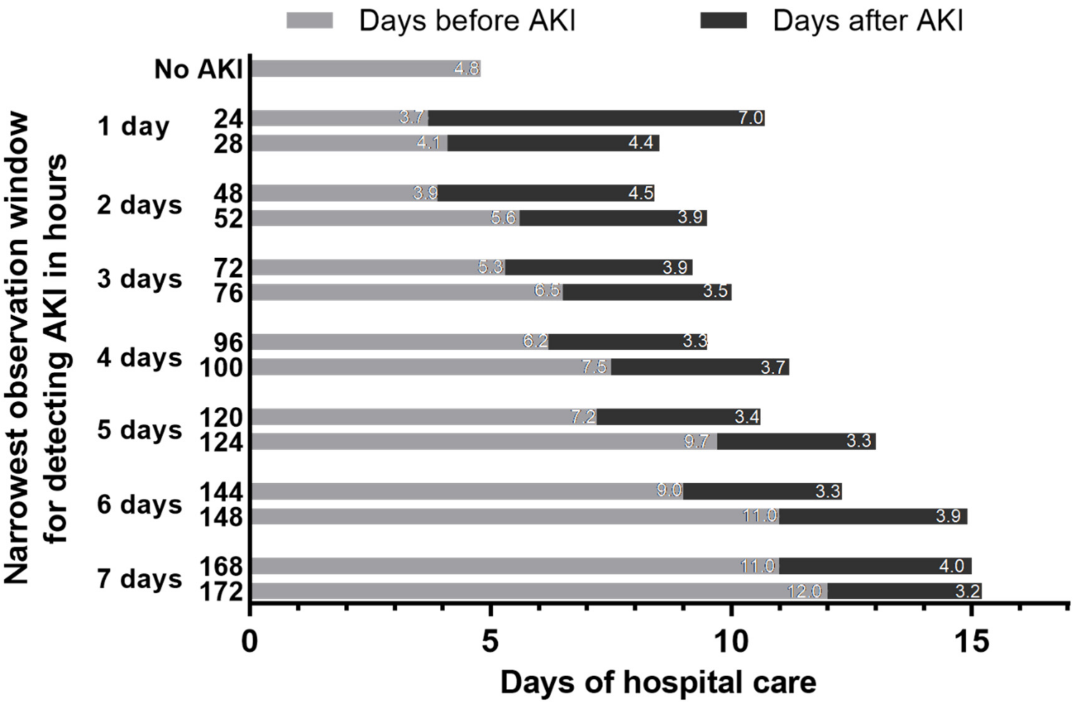

AKI, acute kidney injury.

The conversion factor for units of SCr in mg/dl to µmol/l is x88.4.

Figure S5. Days of hospital care before and after detection of AKI using only criterion 1b (50% relative increase) as the observation window increases from 24 to 172 hours (n=126,367)

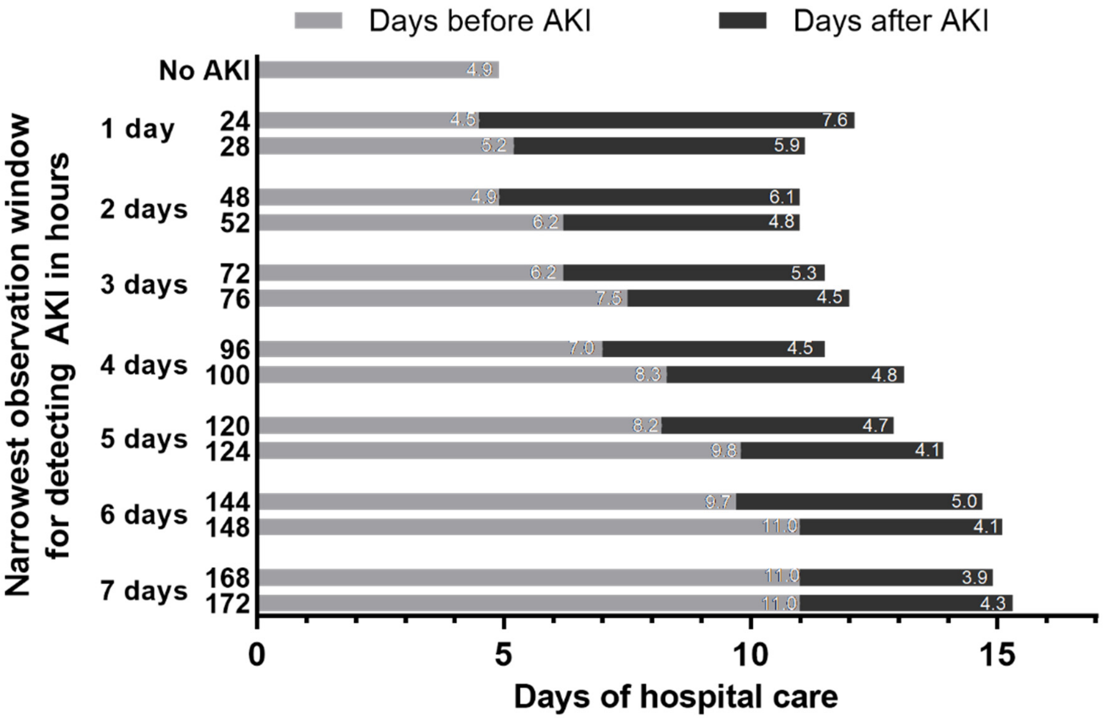

AKI, acute kidney injury.

Figure S6. Adjusted odds ratio of in-hospital mortality by narrowest observation window using only criterion 1a (absolute increase of 0.3 mg/dL)

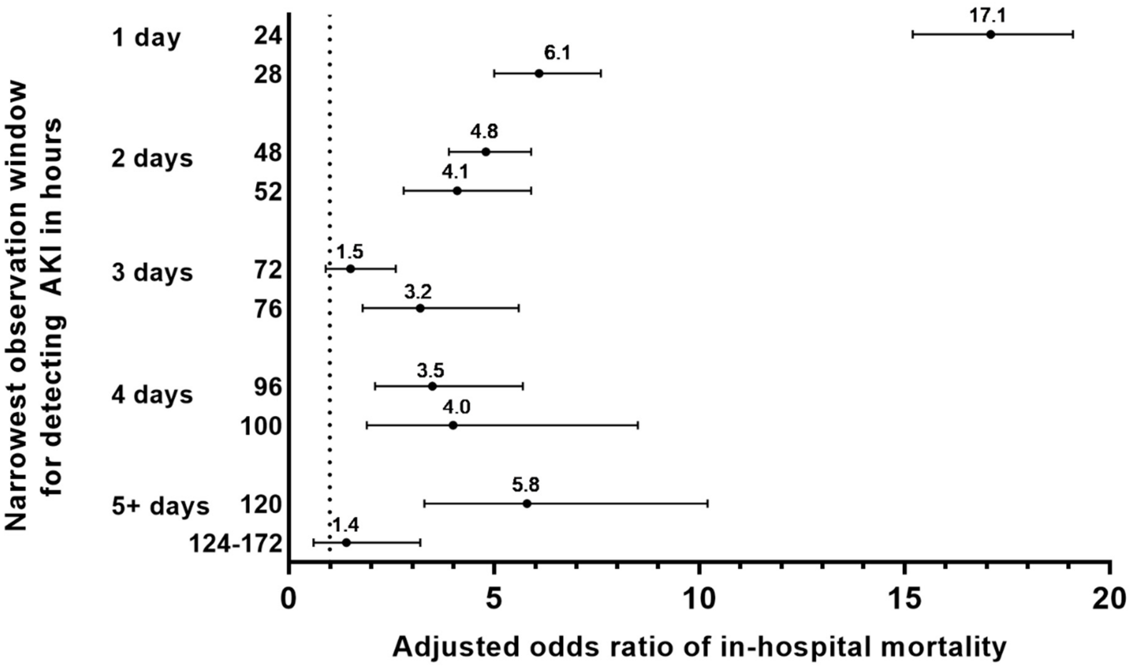

Odds ratios (dots) and 95% confidence intervals (lines) were calculated using logistic regression, where the reference category was patients who did not develop AKI.

AKI, acute kidney injury.

Adjusted odds ratios (dots) and 95% confidence intervals (lines) were calculated using logistic regression, where the reference category was patients who did not develop AKI. Logistic regression was adjusted for hospital admission status (elective/unknown, urgent, and emergency), estimated GFR in categories of 15 ml/min/1.73m^2 based on first hospital serum creatinine, gender, hospital type (academic medical center vs. community hospital), and baseline comorbidities of diabetes, anemia, hypertension, congestive heart failure, liver disease, and peripheral and visceral atherosclerosis. The conversion factor for units of SCr in mg/dl to μmol/l is x88.4.

Figure S7. Adjusted odds ratio of in-hospital mortality by narrowest observation window using only criterion 1b (50% relative increase)

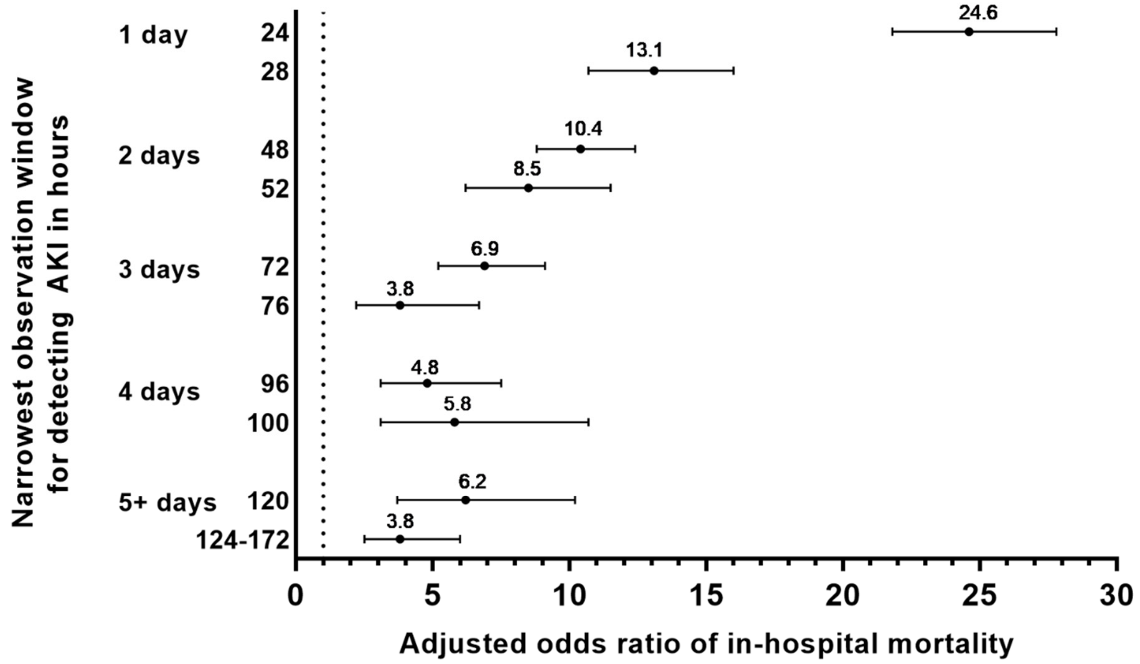

Odds ratios (dots) and 95% confidence intervals (lines) were calculated using logistic regression, where the reference category was patients who did not develop AKI.

AKI, acute kidney injury.

Adjusted odds ratios (dots) and 95% confidence intervals (lines) were calculated using logistic regression, where the reference category was patients who did not develop AKI. Logistic regression was adjusted for hospital admission status (elective/unknown, urgent, and emergency), estimated GFR in categories of 15 ml/min/1.73m^2 based on first hospital serum creatinine, gender, hospital type (academic medical center vs. community hospital), and baseline comorbidities of diabetes, anemia, hypertension, congestive heart failure, liver disease, and peripheral and visceral atherosclerosis.

Table S1. Association between AKI and length of stay (n=126,367)

Narrowest observation window for detecting AKI	n	Adjusted regression with bootstrap sampling[a,b] (n=126,367)	
		Coefficient	95% CI
No AKI	104,473	Reference	Reference
24 hours	9,357	5.5	5.3 to 5.7*
28 hours	3,146	3.4	3.2 to 3.7*
48 hours	3,817	3.4	3.1 to 3.6*
52 hours	1,236	4.3	4.0 to 4.7*
72 hours	1,487	4.2	3.9 to 4.6*
76 hours	596	5.0	4.5 to 5.5*
96 hours	791	4.6	4.2 to 5.1*
100 hours	309	6.1	5.4 to 6.8*
120 hours	419	5.8	5.2 to 6.4*
124 hours	179	7.8	6.6 to 9.0*
144 hours	226	7.8	6.6 to 9.0*
148 hours	117	8.4	7.3 to 9.5*
168 hours	143	9.8	8.4 to 11.1*
172 hours	61	9.7	8.0 to 11.3*

AKI acute kidney injury; CI confidence interval.

* $P<0.001$

[a] Adjusted for hospital admission status (elective/unknown, urgent, and emergency), estimated GFR in categories of 15 ml/min/1.73m^2 based on first hospital SCr, gender, hospital type (academic medical center vs. community hospital), and baseline comorbidities of diabetes, anemia, hypertension, congestive heart failure, liver disease, and peripheral and visceral atherosclerosis.

[b] To evaluate internal validation, the adjusted regression model was replicated 1,000 times using bootstrap sampling.

Table S2. Association between AKI and in-hospital mortality (n=126,367)

Narrowest observation window for detecting AKI	n	Adjusted regression with bootstrap sampling[a,b] (n=126,367)	
		Coefficient	95% CI
No AKI	104,473	Reference	Reference
24 hours	9,357	17.0	15.0 to 19.1*
28 hours	3,146	6.5	5.3 to 8.0*
48 hours	3,817	4.7	3.8 to 5.8*
52 hours	1,236	3.7	2.5 to 5.5*
72 hours	1,487	1.9	1.1 to 3.3
76 hours	596	2.5	1.3 to 5.1
96 hours	791	2.6	1.4 to 4.6
100 hours	309	4.1	1.8 to 9.4*
120 hours	419	4.3	2.2 to 8.5*
124 to 172 hours[c]	726	1.2	0.4 to 3.2

AKI acute kidney injury; CI confidence interval.
* $P \leq 0.001$
[a]Adjusted for hospital admission status (elective/unknown, urgent, and emergency), estimated GFR in categories of 15 ml/min/1.73m^2 based on first hospital serum creatinine, gender, hospital type (academic medical center vs. community hospital), and baseline comorbidities of diabetes, anemia, hypertension, congestive heart failure, liver disease, and peripheral and visceral atherosclerosis.
[b]To evaluate internal validation, the adjusted regression model was replicated 1,000 times using bootstrap sampling. However, one or more parameters could not be estimated in 10 bootstrap replicates, and only 990 replicates were used to estimate standard errors.
[c]Only 5 deaths occurred for patients in narrowest window groups 124, 144, 148, 168, and 172; these were collapsed into a 124- to 172-hour category to improve the stability of the regression model.
